# Remediation of Soil Polluted by Organic Compounds Through Chemical Oxidation and Phytoremediation Combined with DCT

**DOI:** 10.3390/ijerph16173179

**Published:** 2019-08-31

**Authors:** Elena Cristina Rada, Gianni Andreottola, Irina Aura Istrate, Paolo Viotti, Fabio Conti, Elena Romenovna Magaril

**Affiliations:** 1 Department of Theoretical and Applied Sciences, Insubria University of Varese, Via G.B. Vico 46, 21100 Varese, Italy; 2 Department of Civil, Environmental and Mechanical Engineering, University of Trento, via Mesiano 77, 38123 Trento, Italy; 3 Department of Biotechnical System, University Politehnica of Bucharest, Spaiul Independentei 313, sector 6, 060042 Bucharest, Romania; 4 Department of Civil, Constructional and Environmental Engineering, University Sapienza of Rome, Via Eudossiana 18, 00184 Rome, Italy; 5 Department of Environmental Economics, Ural Federal University, Mira Str., 19, Ekaterinburg 620002, Russia

**Keywords:** electroremediation, organic pollution, pelargonium, phytoremediation, TPH

## Abstract

Soils contaminated with organic substances is an important issue across Europe: In some areas, these are the main causes of pollution, or the second after contamination from waste disposal. This paper included an experimental application that compared three methods of remediation of contaminated sites, based on electric fields: A single treatment (electroremediation); and two combined treatments, phyto-electrochemical and electrooxidation (a combination of chemical treatment and a DCT—direct current technology). The contaminated soil was taken from a former industrial area devoted to oil refining, located between two roads: The one national and the other one for industrial use. Nine soil samples were collected at two depths (0.2 and 0.4 m). The initial characterization of the soil showed a density of 1.5 g/cm³ and a moisture of about 20%; regarding grain size, 50% of the soil had particles with a diameter less than 0.08 mm. The electrochemical treatment and electrooxidation had an efficiency of 20% while the two combined methods had efficiencies of 42.5% for electrooxidation (with H_2_O_2_) and 20% for phyto-electroremediation (phyto-ER) with poinsettias.

## 1. Introduction

The issues of soil protection, sustainable use, conservation and remediation (where feasible) have growing importance worldwide, also in view of the environment and human health management [1,2,3,4,5,6]. In the process of harmonization of national policies within the EU and the pathway of transposition and implementation of European rules and regulations, the problem of contamination of soil and groundwater is one of the fundamental aspects of protection of the environment. It should be treated with full responsibility by all the actors involved.

Based on U.S. EPA reports, annually 6 million tons of petroleum pollutants are released into the environment. They put at risk fauna and flora and worsen human health [7,8,9,10]. One of the biggest environmental disasters is the one in the Gulf of Mexico, which seriously affected the ecosystem of the region and human health [11,12,13,14]. In this context, the idea of searching more viable solutions with increasing efficiency becomes more and more important. Therefore, the phytoremediation of petroleum compounds has received more attention by many researchers as an alternative to conventional solutions [15,16,17,18,19,20].

Oil hydrocarbons are among the most common soil and water contaminants in the world [21,22,23]. Petroleum refining industries convert crude oil into more than 2500 petroleum products, including oils, etc. World oil demand is expected to rise to 107 thousand barrels per day over the next two decades, and oil will account for 32% of the world’s energy supply by 2030 [23,24]. Consequently, the environmental issues are central because of the high negative impact associated with aliphatic and aromatic hydrocarbons that have high hydrophobicity, low water solubility, high sorption capacity and high aliphatic solubility [23,24,25,26,27].

Phytoremediation, i.e., the use of plants to reduce the risk associated mostly with heavy metal contaminated soils, has been actively investigated as a low-cost option that causes no deterioration in soil quality [28,29,30]. The idea originated from the identification of hyperaccumulator plants, which can take up large amounts of heavy metals and accumulate high concentrations in their above-ground tissues with no adverse effects on growth [19,29].

Phytoremediation has attracted increasing attention and is a promising technology for addressing soil contamination problems. Unlike other kinds of remediation plants, ornamental plants grown for decorative purposes in gardens and landscape design projects have been an important source of remediation in recent years [31,32,33,34,35]. In addition to beautifying the environment, some ornamental plants also accumulate or degrade contaminants when growing in soil contaminated with heavy metals or organic pollutants [31]. The practical application of phytoremediation is limited to those sites with medium or low contaminant concentration, with sufficient low toxicity to allow the growing of plants. The remediation depth is limited by the plant root depth [36].

Phytoremediation can be applied to soil, water and sediments, with biological, chemical and physical extractions of contaminants with the help of the plants. The plants have a remarkable metabolic structure and the ability to extract nutrients or contaminants from soil [37,38]. Phytoremediation uses mature plants at a contaminated site (for a short period of growth) to remove the contaminants from the soil and to facilitate the isolation or degradation of the pollutants [39]. Subsequently, the plants may be collected, treated and disposed of.

To contrast the wide range of pollutants, the plants have developed a series of genetic changes [40]. It is a passive method suitable for the remediation of sites with moderate or low levels of contamination.

The chemical oxidation is a remediation technology successfully used for the decontamination of soils polluted by hydrocarbons [41,42,43]. It can be applied both in situ and ex situ. In comparison with other reclamation processes, the most beneficial aspect linked to the use of this technology is constituted by the shortness of the time necessary for the application, to which is added the uniformity due to the possibility of continuous mixing of the soil (in the case of application ex situ). However, the cost of ex situ treatment is higher than in the case of the in situ process because of the additional costs for the excavation [44]. The application of the chemical oxidation ex situ involves the mixing of an oxidizing agent with contaminated soil and/or polluted groundwater. The oxidizing agents are the classic ones used for the purification of wastewater (for the treatment of organic contaminants), namely hydrogen peroxide, hypochlorite, chlorine dioxide, potassium permanganate and/or ozone.

In recent years, electrochemical applications used to reduce pollution have received increasing attention [45,46,47,48]. In such applications, the removal of pollutant species takes place directly or indirectly through electrochemical oxidation or a reduction process in the electrochemical cell, without the continuous supply of chemical additives. The selectivity of many electrochemical processes also helps to prevent the production of derivatives and/or waste, which in many cases should be properly treated before disposal [47,48]. Despite these advantages, there are limitations to such applications, for example the heterogeneous nature of the electrochemical processes, as well as the long-term stability and activity of the electrode material and cell components.

Although phytoremediation and electroremediation (ER) are efficient technologies to remove organic pollutants from soil, a significant intensification of the effects could be obtained from the association of them, enhancing the mass transport [23,49]. The combination of phytoremediation and electroremediation has been proposed in an attempt to go over the limitations of phytoremediation [50,51]. The coupled phytoremediation–ER technology consists of the application of a low-intensity electric field to the contaminated soil in the vicinity of growing plants [52]. ER–phytoremediation has already shown very promising results for metals and semimetals.

The application of an electric field near a growing plant may enhance the remediation capacity of the plant by mobilization of nutrients and contaminants that will be more available for plant uptake [23,53,54]. The coupled technology favors the growth of the plant and the microflora associated with the roots, as well as its remedial capacity [55]. Recent research [55] involved the use of *Brassica rapa*, which was selected among 14 other plant species because of its capacity to germinate and grow in mixed contaminated soil. The combined treatment, electro-phytoremediation, with *Brassica rapa* resulted in substantial elimination of polycyclic aromatic hydrocarbons (PAHs). The electricity showed a decisive effect on plant growth and biomass production.

The present study considered two technologies belonging to different categories: Biological and chemical–physical. Combined with the technologies that are based on the use of electric currents, they allow increasing the overall effectiveness of the treatment. The used methodologies are chemical oxidation (using hydrogen peroxide), phytoremediation (using as plants geraniums, poinsettias (Christmas star and *Buxus*) and electroremediation.

*Pelargonium* plants were used in this study. According to the literature, they have a high potential especially for the phytoremediation of metals [29,56]. For the organic compounds, Matinfar et al. [7] tried two types of plants: *Festuca* and *Geranium* (a type of pelargonium). Their results indicated that *Festuca* is capable of total petroleum hydrocarbons (TPH) uptake. Moreover, the TPH content in *Festuca* was higher than the one in *Geranium*. According to this study, both plants can be a potential candidate for TPH uptake from the soil, where the rate of uptake by *Festuca* is higher than that of *Geranium*.

Another type of plant that is mentioned in the literature [23] as a good solution for the implementation of phytoremediation is *Helianthus annuus* (sunflower). It was selected based on previous studies about biomass production and survival capability in mixed contaminated soil with organic and inorganic compounds [23,52]. Furthermore, the sunflower species was reported to be effective in the removal of organic compounds by phytoremediation [23,57]. The use of sunflower resulted in a lower TPH removal when a phytoremediation treatment was used. That indicates that microbial degradation and volatilization of the organic compounds are feasible [23]. Conversely, when phytoremediation was coupled with electroremediation, its efficacy was improved, achieving high TPH removal. That is due to the contribution of the reverse polarity in the mobilization and a decrease in the evaporation flux [23].

## 2. Materials and Methods

### 2.1. Sampling and Soil Characteristics

The soil used for the experimental study was excavated from an ex-industrial area where, for several years, the oil industry processed different products. The contaminated area is situated near the main road on one side and near an industrial road on the other side. For the initial characterization it was decided to take samples from 9 different points at two different depths, 0.2 m and 0.4 m.

The initial characteristics of the soil at time 0 of the treatment were (see Section 2.3 for analytical methods): pH = 8.71 (high enough for the high concentration of hydrocarbons in the soil), ORP = 132 mV (oxidation-reduction potential), an indicator of an oxidation process, EC = 1.46 mS/cm (electroconductivity), E = 680 ohm*cm (electrical resistivity), and TDS = 878 mg/L (total dissolved solids). Additional characteristics are reported in Table 1.

For this research, it was decided to insert the electrodes only at the borders of the contaminated area. This configuration is recommended for laboratory experiments. Also, this was decided because the depth of the contaminated area and also the depth reached by the roots were not very high. The network configuration is usually recommended in a real-scale application.

### 2.2. Treatment Reactors

**Figure 1:**
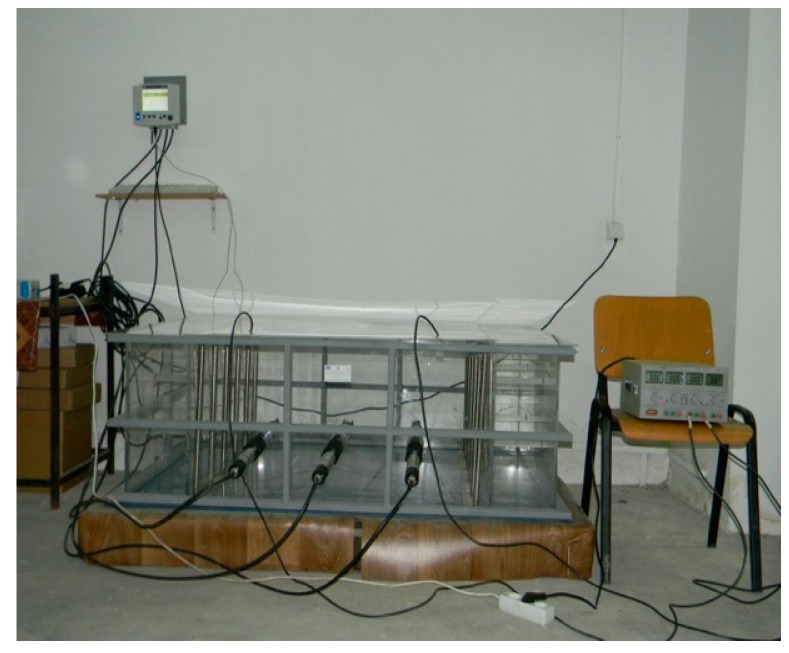
Pilot plant for electroremediation and the electrical and chemical combined treatment.

The main components of the experimental setup for the electroremediation (Figure 1) were: The electrochemical cell, two networks of stainless steel electrodes (an anode and a cathode of 1000 mm × 1000 mm), cables connecting the network of electrodes and the power supply, the power supply (the first system has been applied a voltage of 50 V, while the second 100 V, which corresponds in both cases to a specific minimum voltage equal to 1 V/cm).

**Figure 2:**
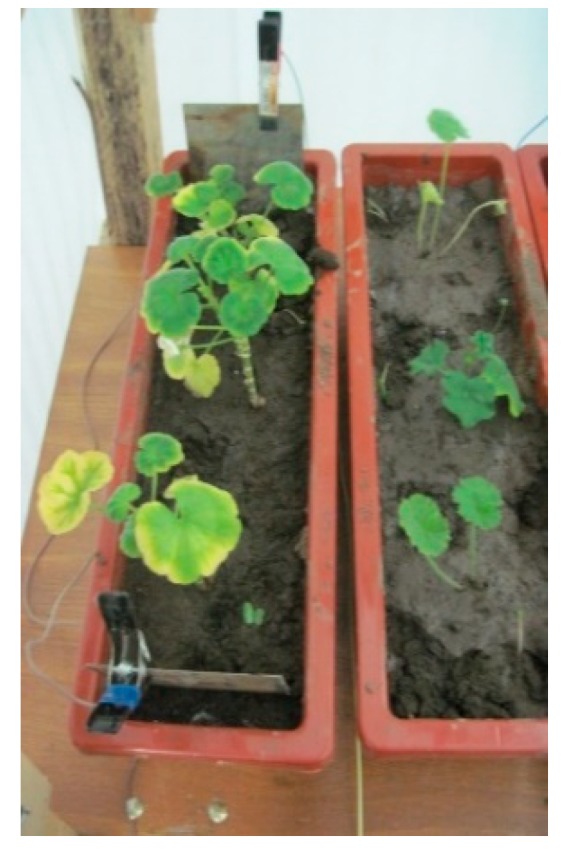
Pilot plant for the application of phyto-electroremediation (phyto-ER).

The pilot plant used for the application of the treatment of phyto-ER of soils contaminated by hydrocarbons (Figure 2) consisted of 2 RGB LEDs, 1 projector (Black 250 W), 1 USB-DMX 512-Pro interface, 4 pots, two power supplies (MCP M10-hp 1502), stainless steel electrodes (rectangular plates of 100 mm × 150 mm), 2 XLR cables pro, and 1 GUI Freestyler.

### 2.3. Analytical Methods

Several parameters were investigated during this study, both to determine the contaminant content and to characterize the main features of the investigated soil. In particular, to perform the study about diesel contaminated soils, the following parameters were measured: Current, pH, temperature, redox potential, soil humidity, soil granulometry and TPH.

The pH, redox and temperature were taken in a soil/water suspension using sensors IQ SENSOR NET pH/ORP, attached to a multiparameter instrument, 2020XT (produced by WTW, Weilheim, Germany). Soil humidity was determined using a dry kiln, where a weighted quantity of wet soil was introduced for 24 h at 105 °C. After this procedure, the dried soil was weighed again and the humidity of soil was determined. Soil granulometry was identified using a series of sieves. The diameters of the sieves ranged from 56 mm to 0.8 mm. The TPH was determined according to the procedure described in SR 13511 (Romanian Standard). For this analysis a SOXHLET (Königswinter, Germany) and a Heidolph Hei VAP Advantage (Schwabach, Germany). The equipment used for these analytical methods has been obtained with the funds from a project co-financed under the Sectorial Operational Programme “Increase of Economic Competitiveness” POSCCE-A2-O2.1.2.-2009-2, RECOLAND ID519, SMIS-CSNR: 11982, Nb. 182/18.06.2010 (2010–2013).

### 2.4. Experimental Tests

The electrochemical technology was used alone for the ER test. The specific voltage was 1 V/cm, which corresponds to an applied voltage of 100 V. The distance between the electrodes was 100 cm. The quantity of the medium to be treated was approximately 550 kg, equivalent to a volume of 0.5 m^3^.

A combined technology, electrochemistry and chemistry, was applied in the test of the electrooxidation. A specific voltage of 1 V/cm was adopted, corresponding to an applied voltage of 100 V. The distance between the electrodes was the same as the previous test and equal to 100 cm, as well as the same was the type of electrodes and the amount of soil. The difference from the previous test is that, in this case, an oxidizing agent was used: H_2_O_2_ at 30%. A total of 6 L was used throughout the treatment: At the beginning of each week, 1 L of hydrogen peroxide was added in the area of the anode and 1 L in the rest of the cell. The initial characteristics of the soil at time 0 of the application were: pH = 8.89, ORP = 75 mV, EC = 159 mS/cm, E = 628 ohm*cm and TDS = 94.5 mg/L.

For the ER test, developed for 110 days, we used two vessels where the plates electrodes were inserted: IPFER 1 with geraniums and IPFER 2 with poinsettias and *Buxus*. We tested three types of plants: geraniums, poinsettias (Christmas star) and *Buxus* (these plants are easy to find and grow in Europe). We added 2 L of water to obtain moisture favorable to the development of the plants and to stimulate the electrochemical processes. For this experiment, 22.07 kg of soil contaminated with hydrocarbons, previously homogenized, were used. The first vessel weighed 11.53 kg and the second 10.54 kg.

From the two vessels, two samples were taken, one for the analysis of hydrocarbons and the other for moisture analysis. No. 7 samples were taken to determine the concentrations of hydrocarbons. During the experimentation, moisture was maintained at 20–25%. Two series of geraniums were planted in IPFER 1 and IPFER 2 together with a series of poinsettias and a *Buxus*. Geraniums and poinsettias were taken from greenhouses managed by the municipal administration, which allowed knowing the age of each plant species. For the first series of plants, the age was about 1–1.2 years: The observed behavior was therefore typical of young plants. The second series of plants had an age of about 3–5 years: The observed behavior was proper to mature plants. In some cases, the onset of diseases such as *Botrytis cinerea* (gray mold), *Xanthomonas geranium* (blackening of the cuttings) and *Agrobacterium tumefaciens* (bacterial cancer) was recorded.

## 3. Results and Discussion

The initial current monitored at the beginning of the experiment was 5.16 A. This was the maximum value reached during the entire experiment, due to the high concentration of hydrocarbons in the soil. After the occurrence of this maximum value, there was a reduction in the current until a constant value. From there, higher values followed in correspondence with the addition of water made to stimulate the electrochemical processes. After these peaks, it decreased to substantially constant levels.

**Figure 3:**
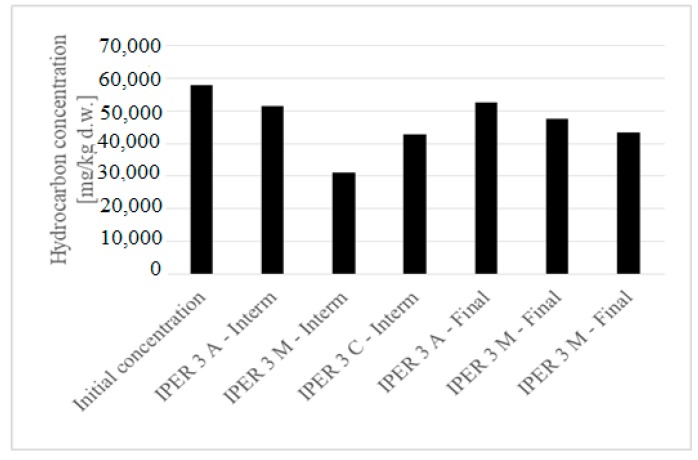
The behavior of hydrocarbons during ER (IPER—Pilot installation for electroremediation).

The planned campaign provided analytical samples at the beginning, at the end and during intermediate moments in the experimental trial. Sampling was carried out in three areas of interest: The anode, the cathode and the center of the sample. The initial concentration of total hydrocarbons was 57,889.48 mg/kg_dw_, about 29 times higher than the threshold for using less sensitive applications (2000 mg/kg_dw_) set by current legislation. The concentrations of hydrocarbons before and after treatment are shown in Figure 3 (average efficiency equal to about 20%).

Since the soil used for the application with ER was highly contaminated, the obtained results were not particularly good, demonstrating the limits of ER in such context. Therefore, it was decided to repeat the test using the same amount of ground, but by combining the electrical treatment with chemical oxidation.

In the test of the electrooxidation, a combined technology was adopted based on electrochemistry and chemistry. The current recorded at the beginning of the experiment was 3 A. During the test, several peaks corresponding to the addition of the oxidant were recorded. The current increased up to 3.8 A. After each peak, the current tended to decrease to a value kept constant until the subsequent addition of oxidizer necessary to stimulate the electrochemical processes.

**Figure 4:**
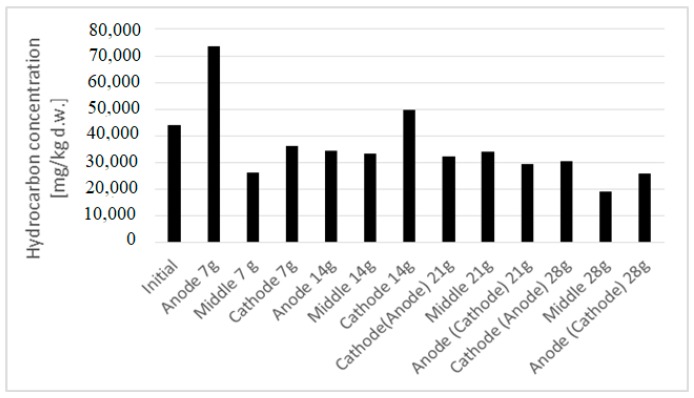
The trend of hydrocarbons during electrooxidation.

After 14 days, since the redox potential was less than 0 in the cathode, it was decided to change the potential, reversing the anode and cathode. At the end of the treatment, after 28 days, an improvement in the results was observed (Figure 4), with a reduction of pollutant concentration of 42.5%. This is the average value of the three zones of interest. At the first intermediate sampling, there was an initial migration of hydrocarbons from cathode and anode to the middle. In the second experimental test, there was a migration of hydrocarbons to the cathode due to the electro-osmotic flux.

For the phyto-electroremediation test, the obtained results are presented in Figure 4. Two samples were taken from the two vessels: One for the analysis of hydrocarbons and the other for moisture analysis. No. 7 samples were taken to determine the concentrations of hydrocarbons. Moisture was maintained at 20–25% during the experimentation. The current in IPFER 1 showed a fluctuation as a function of the amount of water introduced into the system. We could not stabilize a certain value of amperage because of the need for watering the plants every 3–4 days. As a consequence, the current had oscillations during all the 2568 h of experimental testing.

**Figure 5:**
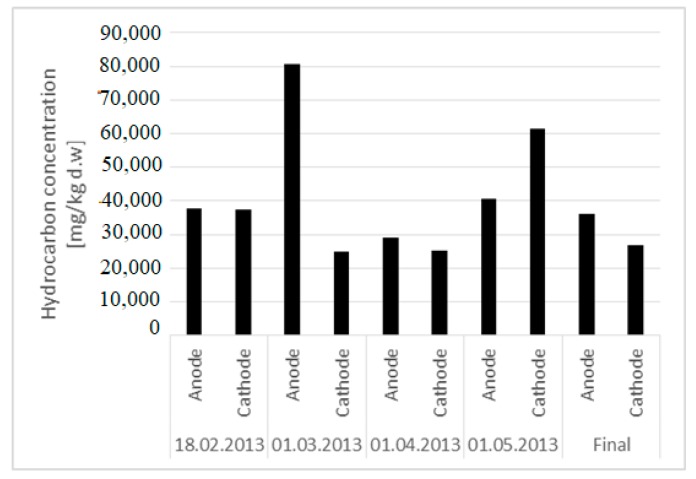
Variation of hydrocarbons during phyto-electroremediation.

The experiments with poinsettias in IPFER 2 showed positive results (Figure 5) and an efficiency of almost 20% as an average value. The results for the geraniums were negative for the obtained yields.

Even though the removal efficiency was around 20%, this method has the advantage that the existing limitations can be better managed with respect to the ones of bioremediation. The pelargonium plant also needs quite a short period for growing. These plants also resist quite well in contaminated soil. Therefore, transplantation of pelargonium plants around fuel stations and sites with possible leakage of oil, diesel fuel and derived compounds is highly recommended.

As a result of the use of electricity in the vessels during phyto-ER, a black dot in the cathode was noticed, which lasted for about three weeks, which was followed by a reduction in diameter. This observation reinforces the idea that the migration of a pollutant to an electrode precedes its reduction for the chemical reactions that take place in the area.

Regarding the combined processes, it has been observed that when the concentrations of the pollutants are too high, in the first phase there is a migration of contaminants between an electrode and the other for the electro-osmotic flux, after which the oxidative and reductive reactions take place. The behavior of the soil under the influence of the electric field is different from what was found in the literature, probably due to the excessively high concentration of hydrocarbons. Since the value of the initial concentration of the pollutant was very high, a clear change in the pH from acid to alkaline zone areas was not observed, as also reported in other similar studies. For this reason, even the ORP had no accentuated variation and the oxidation reactions were not present as much as they would be in order to obtain a high efficiency of treatment. On the other hand, the much larger quantities of contaminated soil should be correlated with the treatment time. To obtain higher efficiency, further experimental tests should be made to apply the treatment for a longer period.

An important aspect, which must be taken into account for future developments of the presented research, is the type of electrodes that are used and also the configuration of them into the soil. That is, a pair of plate electrodes can be used (especially for the laboratory tests), or a couple of tube electrodes which can be distributed in the function of the contamination type, the depth of it and the pollutant type. To also stimulate the growth of plants, the depth of the roots must be considered. For the second type of electrodes the following configuration could be used: The classical anode and cathode networks, or cathode networks on the border of the contaminated soil, and an anode network in the middle.

The results obtained in this research are similar to the ones from other scientific papers [55] where phyto-ER with *Brassica rapa* resulted in substantial elimination of PAHs. The electricity showed a decisive effect on plant growth and biomass production. The results of this work suggest that alternating current (AC) may be the most suitable for large-scale treatment. The spatial distribution of the electrodes in soil has a decisive influence on the distribution of the electric field in soil. For lab applications, the side-by-side scheme is recommended because of the uniform electric field distribution. In field applications, the scheme with a parallel anode and cathode on the soil surface is recommended for its easy implementation [58].

Rocha et al. observed in their research that TPH removal was lower for the phyto-remediation treatment, which indicates that microbial degradation and volatilization of the organic compounds are feasible. Conversely, when phyto-remediation was coupled to electrochemical remediation, its efficacy was improved, achieving high TPH removal due to the contribution of ORP in the mobilization and a decrease in the evaporation flux [23].

## 4. Conclusions

The efficiency of the electrochemical treatments depends on several aspects: Type of soil, initial concentration and type of contaminants, amount of soil to be treated, as well as other factors. Therefore, before application to a real scale, it is preferable to test the technology on a laboratory scale. To this extent, this study described experimental tests on soils contaminated with petroleum products. Specifically, the contaminated soil was taken from a former industrial area devoted to oil refining.

The performed electrochemical treatment and the electrooxidation demonstrated a limited efficiency.

The two combined tested methods showed a significantly higher efficiency for electrooxidation (with H_2_O_2_) and similar results for phyto-ER (with poinsettias).

Even if variable, the obtained results showed to be coherent with the scientific literature of the sector.

The next step of the research will concern the economic issue of the proposed approach as the advances in the field of health and environment must be viable also from this point-of-view.

## Figures and Tables

**Table 1:** Main characteristics of the soil used in the experiments.

Soil Characteristics	Values
Density	1.5 g/cm^3^
Moisture	20%
Soil granulometry	12.78%, d > 4 mm
	3.34%, d > 2 mm
	27.7%, d > 0.8 mm
	56.17%, d < 0.8 mm
